# Use of Immunosuppression, Romiplostim, and Splenectomy to Achieve Remission in a British Shorthair Cat With Primary Immune‐Mediated Thrombocytopenia

**DOI:** 10.1111/jvim.70149

**Published:** 2025-06-17

**Authors:** Josh M. Kennils, Helen E. Wilson

**Affiliations:** ^1^ Langford Vets, Small Animal Referral Hospital, Langford House Langford UK

**Keywords:** ITP, platelets, splenectomy, thrombopoiesis, thrombopoietin

## Abstract

A 2‐year‐old female spayed British Shorthair cat was diagnosed with immune‐mediated thrombocytopenia (ITP) after presenting with a 3‐week history of gastrointestinal bleeding and anemia. Treatment with an immunosuppressive dose of glucocorticoids was initiated, but relapse occurred within 3 weeks. Because of persistent thrombocytopenia and recurrence of clinical signs of active bleeding, despite further treatment with chlorambucil, romiplostim, and subsequently splenectomy and mycophenolate mofetil was used to successfully treat the cat for 6 months. This case describes the first known use of romiplostim for the treatment of ITP in a cat and demonstrates that although the efficacy of sustained romiplostim use might be variable, it might be effective in treating life‐threatening thrombocytopenia while alternative treatments are pursued.

**Abbreviations**
HCT: hematocrit
ITP: immune thrombocytopenia
MMF: mycophenolate mofetil
PLT: platelet concentration
RI: reference interval
TPO‐RA: thrombopoietin receptor agonist
UK: United Kingdom

## 1 Introduction

Primary immune‐mediated thrombocytopenia (ITP) is reportedly rare in cats, with the veterinary literature containing only two small case series and single case reports [1, 2, 3, 4, 5, 6]. The reported prevalence of thrombocytopenia in cats ranges from 1.2% to 5.9% [7, 8, 9]. It has been reported previously that 11% of cases were caused by immune‐mediated disease [9]. Primary ITP is considered a diagnosis of exclusion [10]. Splenectomy is a treatment used occasionally in both human and veterinary patients with ITP considered refractory to medical management, but reports of its efficacy in cats are lacking [6, 11, 12]. Newer treatment options of thrombopoietin receptor agonists (TPO‐RAs) in human medicine include romiplostim and eltrombopag [11, 13, 14]. The use of romiplostim has been described in dogs with primary ITP and other causes of thrombocytopenia, with up to 90% of dogs with primary ITP having improved number of platelets per L (e.g., 151 x 10^9^/L) in one study [15, 16, 17]. Another study demonstrated homology between the amino acid sequence of human and feline thrombopoietin [18], but no reports of the use of TPO‐RAs exist in cats. Herein, we describe the management of ITP in a British Shorthair cat using immunosuppression, romiplostim, and splenectomy.

## 2 Case Description

A 2‐year‐old, indoor only, female spayed British Shorthair cat, weighing 4.8 kg, was referred with a 3‐week history of melena, oral bleeding, progressive lethargy, and inappetence. Physical examination by the referring veterinarian had documented pallor, mild periodontal disease, and a left‐sided pansystolic grade 2/6 heart murmur. A CBC had documented severe non‐regenerative anemia, hematocrit (HCT) 12% (reference interval [RI]: 30.3%–52.2%), and absolute thrombocytopenia, platelet concentration (PLT) 0 /μL (RI: 151–600 x 10^9^L). The cat was treated with meloxicam (0.05 mg/kg PO q24h) and omeprazole (0.5 mg/kg PO q12h) before referral. Six months prior, the cat had presented with hematemesis and inappetence, and absolute thrombocytopenia was also noted on CBC evaluation at that time. The cat had been treated with meloxicam and dietary change, and the clinical signs resolved.

On presentation to our hospital, day 0, the cat was quiet but alert and responsive. Pallor was documented. Cardiac auscultation identified a grade 2/6 left‐sided systolic heart murmur. Generalized mild peripheral lymphadenopathy was identified and there was no evidence of petechiae or ecchymoses. A CBC disclosed a severe, poorly regenerative anemia with HCT of 14% (RI: 27.7%–46.8%), absolute reticulocyte count 80 × 10^9^/L, and severe thrombocytopenia, PLT of 28 × 10^9^/L (RI: 156–626 × 10^9^/L), with no platelet clumps on blood film examination as determined by a board‐certified clinical pathologist on this evaluation and all subsequent occasions of platelet number assessment. Automated hematology results were generated by a Siemens ADVIA 2120 analyzer at a reference laboratory. Serum biochemistry indicated mild hyperglycemia of 122.4 mg/dL (RI: 63–90 mg/dL) and mildly increased serum urea concentration (63.7 mg/dL; RI: 39–63 mg/dL). Coombs test was negative. Feline immunodeficiency virus antibody and feline leukemia virus antigen testing were negative. Serum cobalamin concentration was 620 pmol/L (RI: 231–617 pmol/L). Cytological evaluation of fine needle aspirates of the popliteal lymph nodes was consistent with reactive change and extramedullary hematopoiesis. Echocardiography indicated a left atrial: aortic ratio of 1.5 (> 1.5 is consistent with left atrial enlargement [19]). The cat was sedated for diagnostic imaging using butorphanol, midazolam, and alfaxalone. Computed tomography of the thorax and abdomen documented mild‐to‐moderate generalized lymphadenomegaly and a mild broncho‐interstitial pulmonary pattern. The cat was diagnosed with suspected primary ITP with severe anemia secondary to chronic oral and possibly gastrointestinal hemorrhage.

Serial CBCs documented progressive anemia (HCT, 10%), with no evidence of further regeneration and ongoing severe thrombocytopenia (PLT, 10 × 10^9^/L). The cat received a transfusion of feline packed red blood cells. Immunosuppressive treatment with glucocorticoids was commenced using dexamethasone (0.5 mg/kg IV q24h). A progressively regenerative response was documented 4 days after packed red cell transfusion, consistent with an appropriate bone marrow response. An improved PLT of 146 × 10^9^/L was noted after 6 days of glucocorticoid treatment. The cat was discharged on prednisolone (2.1 mg/kg PO q24h). Table S1 documents selected serial laboratory results.

Chlorambucil (2 mg PO q48h) was initiated in addition to prednisolone on day 37 because of the recurrence of marked thrombocytopenia. The cat subsequently developed hematuria and ecchymoses on both antebrachii. By day 55, these had resolved and a CBC indicated a HCT of 33.6% and PLT of 19 × 10^9^/L, with 0–3 platelets/high power field (hpf) but with evidence of platelet clumps documented on blood film examination, suggestive of adequate platelet mass.

Dexamethasone was commenced (0.4 mg/kg PO q24h) on day 72 because of recurrence of thrombocytopenia, and PO prednisolone was stopped based on a case report in which an improved number of platelets per L (e.g., 151 x 10^9^/L) was observed after such a transition [3]. Transient lethargy and mild hematochezia were reported within 10 days of dexamethasone treatment and resolved without additional treatment. A CBC evaluation on day 92 documented a HCT of 35.4% and PLT of 17 × 10^9^/L, with 0–3 platelets/hpf and small platelet clumps on blood film examination. Further CBC evaluation on days 113 and 141 documented ongoing thrombocytopenia, with no platelet clumps documented on blood film examination.

Thrombocytopenia persisted with no clinical evidence of hemorrhage for 10 weeks after commencement of dexamethasone before ecchymoses on the ventrum and peri‐orbital area were noted. On day 168, a CBC disclosed a PLT of 5 × 10^9^/L and < 1 platelet/hpf documented on blood film examination.

Romiplostim was commenced on day 168 at 5 μg/kg SC (Nplate, 125 microgram powder for solution for injection, Amgen Ltd., UK). The cat was monitored for 24 h post‐injection. Hypotension (70 mmHg) was documented 5 h post‐injection by Doppler, which responded to a 5 mL/kg crystalloid fluid bolus. A CBC 7 days later (day 176) documented a PLT of 203 × 10^9^/L with platelet clumps and macrothrombocytes documented on blood film examination. The subsequent dosing schedule used for the cat is summarized in Table S2. The single‐use romiplostim vial was refrigerated and used for up to 5 weeks as described previously [15]. The cat was anesthetized and underwent splenectomy 46 days after starting romiplostim because of diminished response to treatment. Histopathological evaluation of the spleen identified extramedullary hematopoiesis and marked megakaryocytic hyperplasia (full histopathological details can be found in Supporting Information 3).

A CBC evaluated 3 days post‐splenectomy (day 218) documented a PLT of 4 × 10^9^/L with very small platelet clumps documented on blood film examination. A final 5 μg/kg dose of romiplostim was given, chlorambucil was discontinued, and mycophenolate mofetil (MMF) commenced (10 mg/kg PO q12h). No adverse effects were reported with MMF. Subsequent hematology evaluation was performed using an external reference laboratory by the referring veterinarian which utilizes a Sysmex XN‐V analyzer alongside manual blood film review by a board‐certified clinical pathologist. The number of platelets per L (e.g., 151 x 10^9^/L) increased to 139 × 10^9^/L within 4 weeks of splenectomy and commencement of MMF. Blood film evaluation at this time documented rare schistocytes and moderate numbers of Heinz bodies affecting between 20% and 50% red blood cells. Additional CBCs at 8 and 12 weeks documented PLTs of 11–15 × 10^9^/L with an estimate of 0–3 platelets/hpf. Blood film evaluation documented platelet clumps, consistent with adequate platelet mass and no evidence of Heinz bodies. The cat was receiving dexamethasone (0.4 mg/kg PO q24h) and MMF (10 mg/kg PO q12h) at this time and continued to do so. The cat remained clinically well for 6 months after splenectomy, with no evidence of hemorrhage, and a CBC indicated PLT of 11 × 10^9^/L with 0–3 platelets/hpf and platelet clumps on blood film examination, suggestive of adequate platelet mass.

## 3 Discussion

The diagnosis of primary ITP remains one of exclusion; guidelines have provided an aid to exclude secondary causes of thrombocytopenia before the diagnosis can be made [10]. In our case, appropriate infectious and neoplastic triggers, based on the cat's lifestyle, were excluded before commencement of immunosuppressive treatment. We suspect this cat presented with a non‐regenerative anemia because of chronic, mild, and possibly intermittent oral bleeding, leading to the depletion of iron stores. In light of the presence of absolute thrombocytopenia 6 months before referral, it is suspected ITP was present for at least this duration. After a packed red blood cell transfusion, a regenerative response was observed. Bone marrow disorders are reported as causes of thrombocytopenia in cats [8]. Bone marrow biopsy for cytological evaluation, histology and infectious disease screening was not undertaken in this cat because of a low index of suspicion for such disorders. Screening for *Babesia felis* was not performed because this infection has not been reported in the United Kingdom. Feline hemoplasma testing was not undertaken because of the non‐regenerative anemia and no evidence of hemolysis on blood film evaluation. The diagnosis of ITP in this case was supported by the initial and then sustained response to immunosuppressive treatment after splenectomy.

Medical management of ITP in cats is poorly described, with reported treatments including glucocorticoids, azathioprine, chlorambucil, cyclosporine, intravenous human immunoglobulin, vincristine, enrofloxacin, and doxycycline, with variable degrees of efficacy [1, 2, 3, 4, 5, 6]. Many cats in the aforementioned reports developed diabetes mellitus secondary to prolonged glucocorticoid use [1, 2]. In this case, an initial response to glucocorticoids was short‐lived, with relapse of thrombocytopenia observed soon after discharge with prednisolone monotherapy. The introduction of chlorambucil as a second agent failed to provide a sustained improvement in the cat's PLT. Based on a case report and lack of response, prednisolone was substituted for PO dexamethasone, although no improvement was observed with this substitution [3]. Intravenous human immunoglobulin is reserved for human use only in the UK and so was not available for this case. Chlorambucil was chosen over cyclosporine as a second‐line immunosuppressive drug in this case because of the respective ease of dosing for the patient's body weight. The consensus statement on treatment of ITP in dogs and cats suggests either drug can be used as a second‐line agent in cats and suggests there is insufficient evidence for the recommendation of vincristine [20].

Thrombopoietin plays a major role in platelet production and increased megakaryocyte production in the bone marrow [13]. Romiplostim, a TPO‐RA, is used in human patients with refractory ITP (defined as those who have failed corticosteroid and immunoglobulin treatment and have been splenectomized), or to bridge the gap before splenectomy can be performed [11]. It is administered as a weekly SC injection and can be associated with adverse effects including contusions, epistaxis, upper respiratory tract infections, oropharyngeal pain, vomiting, and fever. Adverse effects often are reported as mild and do not necessitate cessation of romiplostim treatment [14]. Its use has been reported in a case series of five dogs, three with primary ITP and two with secondary ITP with minimal adverse effects and was reported to have sustained PLT for up to 10 months of use [15]. The cat described here experienced hypotension within 4 h of the first romiplostim injection. Hypotension resolved with a crystalloid fluid bolus and no further adverse events were experienced with subsequent injections of romiplostim. Hypotension is reported as an uncommon adverse effect of romiplostim in the package insert, but the mechanism of hypotension secondary to romiplostim administration has not been reported.

In a case series of 5 dogs, doses of romiplostim ranged from 3 to 13 μg/kg once weekly, with doses of 3–5 μg/kg once weekly used in four of the five dogs. Higher doses of 10–13 μg/kg were required in one dog with ITP secondary to ehrlichiosis [15]. In a retrospective study of 48 dogs with naturally occurring thrombocytopenia of various causes treated with romiplostim, treatment was more effective in dogs with primary ITP as opposed to thrombocytopenia of other causes (e.g., infectious, disseminated intravascular coagulopathy, chemotherapy‐induced) [17]. In the case described here, romiplostim doses of 5–10 μg/kg were utilized, with an adequate number of platelets per L (e.g., 151 x 10^9^/L) maintained for the first 3 weeks at a dose of 5 μg/kg. Increasing doses of romiplostim subsequently appeared to increase PLT, initially for 7 days (as observed on day 197), and then for a shorter duration (as seen on day 215), with a relapse of thrombocytopenia observed by 7 days post‐injection (as seen on day 219). The reason for such treatment failure is unclear. In human medicine, rare treatment failures have been associated with formation of anti‐TPO antibodies, although even a short‐lived response would not have been expected if that were the case here [13]. Measurement of anti‐TPO antibodies was not possible in this case. Dose‐dependent responses are seen in humans, as we observed in the cat reported here, with higher doses resulting in higher PLT [21]. The same vial of romiplostim was used for up to 5 weeks; it did not appear that storage of romiplostim led to treatment failure because a higher dose of a stored vial (e.g., 7 μg/kg) used on day 190 led to recovery of the number of platelets per L (e.g., 151 x 10^9^/L) by day 197. Although considered unlikely, it is possible platelet clumps were not visualized during romiplostim treatment and that adequate mass was still present.

Splenectomy has been reported as an adjunctive treatment for ITP in both human and veterinary patients. A recent study documented complete remission of ITP in 3/7 dogs after splenectomy, with documentation of number of platelets per L (e.g., 151 x 10^9^/L) within the reference interval achieved within 14 days of surgery [12]. A case report described the use of splenectomy as an adjunctive treatment for primary ITP in a cat. Recovery from thrombocytopenia was reported after splenectomy, but relapse occurred as concurrent glucocorticoid treatment was withdrawn [6]. A CBC performed 3 days after splenectomy in the cat described here indicated persistence of thrombocytopenia. After splenectomy, MMF was commenced, with recovery of PLT observed 2 weeks later. We suspect the improvement noted at this time was due to MMF, although a combination of splenectomy and additional immunosuppression is possible. Short‐term PO MMF is reportedly well tolerated and safe in cats at dosages ≤ 15 mg/kg q12h; self‐limiting diarrhea might develop at higher doses [22]. In this case, the cat tolerated MMF without adverse effects for over 6 months. Mycophenolate mofetil was reported to be beneficial within 7 days in two cats with immune‐mediated hemolytic anemia when used as an adjunctive agent, hence this drug being chosen over cyclosporine [23]. Interestingly, increased percentages of Heinz bodies were observed on blood film evaluation post‐splenectomy. Changes in red blood cell morphology, specifically the observation of Heinz bodies and Howell‐Jolly bodies, have been reported in humans with hyposplenism or post‐splenectomy, but such findings have not been reported in veterinary patients [24, 25]. Increased numbers of Heinz bodies can be observed in cats with no clinical relevance, and the cat reported here had no observable adverse effects as a result of the Heinz bodies [26].

In conclusion, we describe the treatment of primary ITP in a cat using romiplostim and splenectomy alongside immunosuppression with glucocorticoids and initially chlorambucil and subsequently MMF. Although romiplostim did not appear to provide long‐term remission with sustained use, as previously described in humans and dogs, given the rapid response to such treatment, it might be of use in emergency situations, or before surgery to decrease the risk of hemorrhage. Hypotension was the only noted adverse effect of its use, observed on a single occasion out of 9 romiplostim doses administered, and monitoring of blood pressure in cats receiving romiplostim therefore is suggested. The cat's platelet mass appeared adequate for up to 6 months after MMF treatment, suggesting this drug may be of use as an alternative immunosuppressant in the management of ITP in cats.

## Disclosure

Authors declare no off‐label use of antimicrobials.

## Ethics Statement

Authors declare no institutional animal care and use committee or other approval was needed. Authors declare human ethics approval was not needed.

## Conflicts of Interest

The authors declare no conflicts of interest.

## Supporting information


**Data S1.** Supporting Information.

**Table S1.** Summary of laboratory results obtained during the cats’ initial treatment period. Day 0 represents the day on which the cat was admitted to the hospital.

**Table S2.** Summary of automated number of platelets per L (e.g., 151 × 109/L), blood film examination findings and dose of romiplostim used. All results obtained using hematology analyzer Siemens ADVIA 2120.
