# Supplementary material for: Use of Immunosuppression, Romiplostim, and Splenectomy to Achieve Remission in a British Shorthair Cat With Primary Immune‐Mediated Thrombocytopenia
Source: J Vet Intern Med. 2025 Jun 17;39(4):e70149. doi: 10.1111/jvim.70149 (PMC12172128; doi:10.1111/jvim.70149)
Supplement: Supplementary file 1 — Data S1. Supporting Information. [file JVIM-39-e70149-s003.docx]

Supplementary Material 3.

Histopathological evaluation of the spleen:

Histological description: Splenic parenchyma shows irregular congestion with multiple foci of extramedullary hematopoiesis comprising large numbers of megakaryocytes. These megakaryocytes are large to giant with irregularly lobated nuclei that often present with condensed chromatin. Some of the megakaryocytes present with a more basophilic cytoplasm and higher nucleus-cytoplasm-ratio. Periarterial lymphatic sheets appear mildly expanded.

Histological diagnosis: Congestion with extramedullary hematopoiesis with megakaryocytic hyperplasia, marked, spleen.
