# Supplementary material for: Use of Immunosuppression, Romiplostim, and Splenectomy to Achieve Remission in a British Shorthair Cat With Primary Immune‐Mediated Thrombocytopenia
Source: J Vet Intern Med. 2025 Jun 17;39(4):e70149. doi: 10.1111/jvim.70149 (PMC12172128; doi:10.1111/jvim.70149)
Supplement: Supplementary file 2 — Table S1. Summary of laboratory results obtained during the cats’ initial treatment period. Day 0 represents the day on which the cat was admitted to the hospital. [file JVIM-39-e70149-s001.docx]

| Parameter (reference interval) | Day 0 | Day 1 | Day 3 | Day 6 | Day 8 | Day 10 | Day 16 | Day 35 | Day 37 | Day 55 | Day 72 | Day 92 | Day 113 | Day 141 | Day 168 |
| --- | --- | --- | --- | --- | --- | --- | --- | --- | --- | --- | --- | --- | --- | --- | --- |
| Hematocrit (27.7-46.8%) | 14.4 | 10.3 | 10.5 | 13.1 | 13 | 15.5 | 24.7 | 33.5 | 28.6 | 33.6 | 32.8 | 35.4 | 36.1 | 33.8 | 30.1 |
| Reticulocytes (x10^12^/l) | 0.08 | 0.05 | 0 | 0.1 | 0.27 | 0.4 | 0.03 | 0.01 | n/a | 0.02 | 0.01 | 0.05 | 0.01 | 0.02 | 0.09 |
| Platelets (156-626 x10^9^/l) | 28 | 10 | 10 | 146 | 78 | 136 | 11 | 12 | 8 | 19 | 12 | 17 | 8 | 7 | 5 |
| White blood cells (6.3-19.6 x10^9^/l) | 6.99 | 5.44 | 9.32 | 36.7 | 15.26 | 18.13 | 11.4 | 15.2 | 11.04 | 9.6 | 11.5 | 15.4 | 11.1 | 10.4 | 9.42 |
| Neutrophils (3-13.4 x10^9^/l) | 3.84 | 3.43 | 6.34 | 32.66 | 13.12 | 13.96 | 7.02 | 12.63 | 8.86 | 7.20 | 9.71 | 12.43 | 9.61 | 9.20 | 8.35 |
| Albumin (2.4-3.5 g/dL) | 2.7 |  |  |  |  | 3.0 |  |  |  |  |  |  |  |  | 3.3 |
| Urea (39-63 mg/dL) | 63.7 |  |  |  |  | 35.4 |  |  |  |  |  |  |  |  | 47.4 |
| Total bilirubin (58.5-497 mg/dL) | 64.3 |  |  |  |  | 58.5 |  |  |  |  |  |  |  |  | N/A |
| Smear | 1 Plt/ hpf | <1 Plt/ hpf | <1 Plt/ hpf | Platelets plentiful | Platelets plentiful | Platelets plentiful | Platelet clumps seen | <1 Plt/ hpf | <1 Plt/ hpf | 0-3 Plt/ hpf. Platelet clumps seen | 0-3 Plt/hpf. Very small platelet clumps seen | 0-3 Plt/hpf. Very small platelet clumps seen | <1 Plt/ hpf | <1 Plt/ hpf | <1 Plt/ hpf |
| Additional comments |  | pRBC transfusion after this and glucocorticoid therapy started |  |  |  |  |  |  | Chlorambucil therapy started |  |  |  |  |  | Romiplostim started after this |

Supplementary Table 1: Summary of laboratory results obtained during the cats’ initial treatment period. Day 0 represents the day on which the cat was admitted to the hospital.
