# Supplementary material for: Use of Immunosuppression, Romiplostim, and Splenectomy to Achieve Remission in a British Shorthair Cat With Primary Immune‐Mediated Thrombocytopenia
Source: J Vet Intern Med. 2025 Jun 17;39(4):e70149. doi: 10.1111/jvim.70149 (PMC12172128; doi:10.1111/jvim.70149)
Supplement: Supplementary file 3 — Table S2. Summary of automated number of platelets per L (e.g., 151 × 109/L), blood film examination findings and dose of romiplostim used. All results obtained using hematology analyzer Siemens ADVIA 2120. [file JVIM-39-e70149-s002.docx]

| Day | Automated PLT at time of injection (x10^9^/L) | PLT per high power field | Clumps on blood film examination? | Macrothrombocytes on blood film examination? | Platelet count considered adequate? | Dose of romiplostim administered (µg/kg) | Additional comments |
| --- | --- | --- | --- | --- | --- | --- | --- |
| 168 | 5 | <1 | No | No | No | 5 | Petechiae and ecchymoses present |
| 176 | 203 | Numerous | Yes | Yes | Yes | 5 | Petechiae and ecchymoses resolved |
| 183 | 5 | Numerous | Yes | No | Yes | 5 |  |
| 190 | 2 | <1 | No | Yes | No | 7 |  |
| 197 | 30 | Numerous | Yes | Yes | Yes | 6 |  |
| 204 | 2 | <1 | No | No | No | 8 |  |
| 211 | 3 | <1 | No | Yes | No | 10 |  |
| 215 | 22 | Numerous | Yes | No | Yes | N/A | Splenectomy performed |
| 218 | 4 | Subjectively reduced | Small clumps in tail of smear | No | No | 5 | Chlorambucil stopped and mycophenolate mofetil started |

Supplementary Table 2: Summary of automated platelet count, blood film examination findings and dose of romiplostim used. All results obtained using hematology analyzer Siemens ADVIA 2120
